# Measuring Health System Strengthening: Application of the Balanced Scorecard Approach to Rank the Baseline Performance of Three Rural Districts in Zambia

**DOI:** 10.1371/journal.pone.0058650

**Published:** 2013-03-21

**Authors:** Wilbroad Mutale, Peter Godfrey-Fausset, Margaret Tembo Mwanamwenge, Nkatya Kasese, Namwinga Chintu, Dina Balabanova, Neil Spicer, Helen Ayles

**Affiliations:** 1 University of Zambia School of Medicine, Department of Community Medicine, Lusaka, Zambia; 2 Clinical Research Department, Faculty of Infectious and Tropical Diseases, London School of Hygiene and Tropical Medicine, London, United Kingdom; 3 ZAMBART Project, Ridgeway Campus, University of Zambia, Lusaka, Zambia; 4 Department of Global Health and Development, Faculty of Public Health and Policy, London School of Hygiene and Tropical Medicine, London, United Kingdom; Tulane University School of Public Health and Tropical Medicine, United States of America

## Abstract

**Introduction:**

There is growing interest in health system performance and recently WHO launched a report on health systems strengthening emphasising the need for close monitoring using system-wide approaches. One recent method is the balanced scorecard system. There is limited application of this method in middle- and low-income countries. This paper applies the concept of balanced scorecard to describe the baseline status of three intervention districts in Zambia.

**Methodology:**

The Better Health Outcome through Mentoring and Assessment (BHOMA) project is a randomised step-wedged community intervention that aims to strengthen the health system in three districts in the Republic of Zambia. To assess the baseline status of the participating districts we used a modified balanced scorecard approach following the domains highlighted in the MOH 2011 Strategic Plan.

**Results:**

Differences in performance were noted by district and residence. Finance and service delivery domains performed poorly in all study districts. The proportion of the health workers receiving training in the past 12 months was lowest in Kafue (58%) and highest in Luangwa district (77%). Under service capacity, basic equipment and laboratory capacity scores showed major variation, with Kafue and Luangwa having lower scores when compared to Chongwe. The finance domain showed that Kafue and Chongwe had lower scores (44% and 47% respectively). Regression model showed that children's clinical observation scores were negatively correlated with drug availability (coeff −0.40, p = 0.02). Adult clinical observation scores were positively association with adult service satisfaction score (coeff 0.82, p = 0.04) and service readiness (coeff 0.54, p = 0.03).

**Conclusion:**

The study applied the balanced scorecard to describe the baseline status of 42 health facilities in three districts of Zambia. Differences in performance were noted by district and residence in most domains with finance and service delivery performing poorly in all study districts. This tool could be valuable in monitoring and evaluation of health systems.

## Introduction

There is growing interest in health system performance and recently WHO launched a report on health systems strengthening emphasising the need for close monitoring using systems wide approaches [1], [2], [3]. This has been driven by the demand for performance improvement based on efficient use of limited resources in the presence of overwhelming health needs. Different approaches and methods have been used to measure health system performance, especially in high-income countries [3], [4]. The WHO and the OECD, for example, have compared and ranked health systems across a range of functions and performance indicators. These exercises have sometimes been controversial but also difficult to achieve because of the complexity of comparing different health systems [5], [6], [7].

Health service planners and managers are faced with numerous challenges, not least having limited resources with which to provide services at an acceptable level of quality that are equitable and accessible to all. In order to monitor performance of interventions, various attempts have been made and one fairly recent method is the use of the balanced scorecard (BSC) system. The balanced scorecard is derived from the private business ‘balanced scorecard’ approach, a strategic management tool that was first suggested by Robert Kaplan and David Norton in 1992 [8]. The idea is that a scorecard provides information on areas of strategic importance to guide future planning, but also serves as a snapshot of how well an organization or system is performing [7]. A balanced scorecard is made up of domains and indicators derived from the strategic vision of an organisation aimed at measuring its performance. The design and implementation of the balanced scorecard process can be separated into four stages: (1) translating the vision and gaining consensus; (2) communicating the objectives, setting the goals, and linking strategies; (3) setting targets, allocating resources, and establishing milestones; (4) feedback and learning. [9], [10]. Originally the balanced scorecard approach was based on four different perspectives of equal weight: learning and growth, internal processes, customer satisfaction, and financial performance. However, when applied to the healthcare sector, the four traditional perspectives needed further modification to better reflect the particular functions of the public health sector [11]. Balanced scorecards have been used in healthcare monitoring and evaluation at patient, facility, district and national level but mostly in high income countries [12]. The WHO endorsed the balanced scorecard approach in evaluating health system strengthening interventions in low income countries [13]. One study conducted in Afghanistan used the balanced scorecard approach to evaluate the performance of the health system based on selected indicators over a period of five years. In this work Edward et al, (2011) made important modifications to the traditional balanced scorecard. They included domains such as patient and community, human resources, service provision and health system preparedness indicators for equipment, essential commodities and infrastructure [9], [14]. We adapted and applied the BSC approach in the context of the Zambian health care system. The Zambian health system is comprised of 9 Provincial Health Offices, 72 District Health Offices, 98 hospitals, 265 urban health centers, 1,029 rural health centers, and 171 health posts. Health centers are intended to serve 30,000 to 50,000 people in urban areas and 10,000 people in rural areas, within a 29 kilometer radius catchment area. Human resource challenges for the health sector in Zambia are well documented [15]. Shortage of skilled health workers constitutes a very important bottleneck to service delivery. According to records from Ministry of Health (MOH), the total number of staff in the health sector stands at 29,533, this is 57 percent of the approved establishment. Less than 50% of frontline health workers (nurses, midwives, clinical officers, Environmental Health Technicians, (EHT)) are available in relation to the need [16].

Public health facilities in rural and remote areas have the lowest number of health workers compared to urban areas [16]. The result is that there are a number of Health Posts and Rural Health Centres which are run by unqualified staff or have only one qualified staff [15], [16]. Other major bottlenecks in health service delivery include weak health infrastructure, inadequate drugs and medical supplies and poor funding. These have been captured as the major focus areas for the MOH 2011 strategic plan [16].

The Better Health Outcome through Mentoring and Assessment (BHOMA) project is a randomised step wedged community trial that aims to strengthen health systems in three Lusaka districts. Before the implementation of the BHOMA intervention, a baseline study was undertaken to determine the baseline characteristics of all the health facilities taking part in the study. We adapted the domains by Edward et al, (2011) to describe the baseline status of all participating health facilities in line with the vision of the Zambian MOH as articulated in the Strategic Plan of 2011 [16].

## Methodology

The BHOMA project targets to strengthen the health system in Chongwe, Kafue and Luangwa covering 48 health facilities (6 pilot sites and 42 intervention sites). The combined population for the three districts is 306,000. Two of the health facilities included in the BHOMA study are affiliated to mission hospitals. These are Katondwe and Mphanshya health facilities which act as outpatient departments for the respective hospitals. Mphanshya mission hospital is in Chongwe district. It has a bed capacity of 90, while Katondwe mission hospital is the main referral hospital in Luangwa district with a bed capacity of 80. All the mission hospitals are well staffed and funded with the help from Churches Association of Zambia (CHAZ). They all offer inpatient and outpatient services, laboratory and X-ray services. Therefore the hospital affiliated health facilities are well supported in terms of staffing and resources compared to other rural health facilities.

The BHOMA model is made up of three primary strategies designed to work at different levels of the health system. These are District, health facility and community strategies. Following is a summary description of the three BHOMA strategies.

### The district strategy

Each of the three districts has one Quality Improvement (QI) team that implements the intervention in target health facilities. Each QI team consists of two nurses and one clinical officer. The teams work closely with the district clinical care specialist who represents the interest of the Ministry of Health. The district QI team is supported by the central Quality Improvement team that provides technical and logistical support to the district teams. The district team implements the intervention in target health facilities in line with the predetermined randomised step wedged design. At the health facility, the QI team works intensively with local clinic staff to build clinical skills, applying clinical protocols and algorithms, completing forms, and reviewing patients together. They work one-on-one to mentor about good patient consultation, ordering appropriate investigations, interpreting results, and working through diagnoses.

### The health facility strategy

The health facility-based intervention aims to improve clinical care quality by implementing practical tools that establish clear clinical care standards, providing essential resources to meet these standards and communicating standards through intensive clinic implementations. Each clinic generating self assessment reports that help identify areas of weakness for further improvement with support from the QI team. Leadership training is provided to the health workers targeting governance, finance, supply chain and human resource management. Staffing support consists of community workers trained as “Clinic Supporters.” These lay workers are trained to assume as many non-clinical duties as possible. These include registration of patients, filing, triaging, recording vital signs, fast tracking urgent cases and routing patients through services.

### The community strategy

The BHOMA project has engaged community health workers on part time basis each earning about $60 per month. They are trained in providing preventive services and tracking missed clinic appointments. They work in collaboration with community health units known as Neighbourhood Health Committees (NHCs) and Traditional Birth Attendants (TBAs).The community health workers are also being trained in capturing and recording local health data and sending it to health facilities via mobile phones or physically. In order to ensure objective evaluation, the BHOMA study has a separate evaluation team.

The evaluating team is composed of health systems experts, epidemiologists and anthropologists. There is a close collaboration between the implementation and the evaluation teams.

### Health facility survey

A baseline health facility survey was conducted in 42 out of 48 health facilities found in the three BHOMA districts. This constituted 96% of the total health facilities with the rest being used as pilot sites for the BHOMA intervention. The study was conducted between January and May 2011. At each health facility a number of questionnaires were administered, targeting health facility managers, health workers and patients. At each health facility the health facility incharge was interviewed, in addition to two other health workers. At each health facility, five adult observations were done irrespective of the presenting complaint. Children were observed if they were under five years and presenting with fever, cough or diarrhoea. Similarly, five exit interviews for adults and five for under five child/guardian pair were done. The recruitment was consecutive until the required number was reached. (See Table 1)

**Table 1:** Sample profile for the baseline study in the three BHOMA districts.

Sample profile		Baseline number
Districts		3
Facilities		42
Patient observations:		
	*Children*	202
	*Adults*	208
**Total**		**410**
Exit Interview		
	*Children*	209
	*Adults*	220
**Total**		**429**
Health provider interviews		96

The selection of indicators was done in three stages. Firstly, available tools and indicators from WHO, Measure Evaluation Facility Surveys and Health Facility Assessment Network (HFAN) were reviewed. Relevant indicators to the domains of interest were selected some of these have been used in previous health facility surveys in Zambia. In the second place, consultations were held with the district and health facility managers to review the indicators and agree on which ones best would address the domains of interest. The tools and indicators were then pre-tested in pilot sites within the BHOMA intervention area and adaptations were made based on pre-test experience. Verbal responses were validated through inspection and physical observation.

Data collection was conducted by the evaluating team composed of a team leader who is a medical doctor and an epidemiologist and fifteen sixth year medical students who were research assistants. Data collectors were trained for five days on how to administer the study tools. Main hospitals and private clinics were excluded from the study. However, hospital affiliated health facilities were included. Health posts were considered as part of the health facility to which they referred patients. Appointments were made with health facility managers prior to the day of data collection.

### Household survey

A household survey was conducted in a random sample of 120 households which fell under respective target health facilities. Households were eligible for inclusion in the study if they had any person above 18 years of age. The households were enumerated and a standardised questionnaire based around validated demographic and health indicators from the Demographic and Health Survey (DHS) were used. In addition, questions were asked about health seeking behaviour, key coverage indicators for both adult and children. A total of 39,012 respondents were approached to take part in the survey. 246 refused to take part giving a refusal rate of 0.6%. The full methodology of the BHOMA intervention is described elsewhere [17].

### Data analysis

Data were entered onto an Access database and exported to SPSS version 19 for analysis. Simple frequencies were used to analyse and explore the data. Comparisons were made between health facilities and districts based on the modified balanced scorecard approach. The analysis utilized indicators reflecting the 2011 MOH Strategic Plan. These were: Service delivery (availability and quality); human resources (motivation and training); finance (availability of action plans and training); service capacity (basic infrastructure, basic equipment, laboratory capacity, tracer drugs and infection control). Patient perspectives were elicited through exit interviews and clinical observations. Gender differences in service satisfaction were used to assess equity of access as reflected in the vision of the Ministry of Health in Zambia. This was taken as a proxy to overall vision as required when applying balanced scorecard approach. In addition, we calculated coverage scores for 6 indicators of access to health services (3 for adult and 3 for child health) (Table 2). We applied linear regression to establish correlations between the different domains of health service delivery.

**Table 2:** Summary of indicators used to calculate Service coverage score in the household survey.

	n	(%)
1. Children with diarrhoea in the last two weeks and seeking treatment	190	42.4
2. Children with cough in the last two weeks and seeking treatment	190	76.6
3. Children with fever in the last two weeks and seeking treatment	258	73.9
4. Adults with high blood pressure on treatment	680	28.7
5. Adults with HIV and on ART	1032	75.9
6. Women on some form of contraception	5037	52.5

### Ethical considerations

The study was approved by the University of Zambia Bioethics Committee and the London School of Hygiene and Tropical Medicine Ethics Committee. All respondents were informed about the purpose of the survey and were asked to sign a consent form before taking part in the study. Confidentiality was ensured during data collection and subsequent publication of the results.

## Results

### Characteristics of sampled health facilities

Forty two health facilities were included in the sample: 21 in Chongwe, 14 in Kafue and 7 in Luangwa district. Nineteen percent (8/42) of the health facilities were classified as peri-urban, while 78.5% (32/42) were classified as rural. Five percent (2/42) were attached to a mission hospital and served as outpatients departments. Twenty one percent of the health facilities had no overnight bed capacity. Fifty percent of the health facilities had at least 6 health workers, some of whom were not formally trained but assisted in reviewing patients. All of the health facilities had a private room for examining patients with visual and audio privacy. Most health workers used their own mobile phone for communication in their work place (57%) with less than 35% having access to work place communication facilities.

Seventy three percent of the facility managers said they did not have access to emergency ambulance services. The majority of the facilities had access to power either through electricity (54.8%) or solar energy (31%). Out of those who said they had access to power about 20% said it was not functional on the day of data collection. Most health facilities had access to safe drinking water and improved type of pit latrines. (See Table 3).

**Table 3:** Baseline demographic characteristics of the health facilities in the BHOMA study.

Variable		n	%
**Residence**			
	Peri urban	8	19.0
	Rural	32	78.5
	Hospital	2	5.0
**Bed capacity**			
	No overnight bed	9	21.4
	1–3 beds	7	16.7
	4–5 beds	7	16.7
	6+ beds	19	45.2
**Number of health workers**			
	Two	5	11.9
	Three	3	7.1
	4–5	13	31.0
	Six plus	21	50.0
**Private consultation room**			
	Yes	42	100.0
**Phone availability**			
	No	4	9.5
	Yes	14	33.4
	Use personal mobile phones	24	57.1
**Access to ambulance**			
	No	31	73.8
	Yes, Functional with fuel	10	23.8
	Yes, not functional	1	2.4
**Power Source**			
	No	5	11.9
	Electricity	23	54.8
	Solar energy	13	31.0
	Generator	1	2.3
**Power working today**			
	Yes	30	81.1
	Not functional	7	18.9
**Water source**			
	Safe protected Source	41	97.6
	Unprotected source	1	2.4
**Toilets for clients**			
	No toilet	1	2.3
	Yes Improved pit latrines	40	95.4
	Flush toilet	1	2.3
**Condition of toilet**			
	Functional	40	97.6
	Not functional	1	2.4

### Patient domain

The patient domain had separate satisfaction indices for children and adults (Tables 4 and 5). Overall adults' satisfaction scores were higher than children's scores (based on parent/guardian responses). Children satisfaction scores ranged between 58% and 65%, while adult scores ranged between 70% and 76%. Children's satisfaction scores were lowest in Kafue (58%) and highest in Chongwe (65%). The highest score for adult satisfaction index was in Luangwa (76%) and lowest in Kafue (70%).

**Table 4:** Baseline District Performance in Six Health System Domains.

Domain	Districts scores(mean)		
Domain A: Patients and community:	Chongwe	Kafue	Luangwa
Patient satisfaction children index	65.4	58.3	61.9
Patient satisfaction Adult index	72.9	70.1	76.3
Service coverage Children index	71.8	73.	76.4
Service coverage Adult index	51.0	52.5	57.1
**Domain B: Human resources**			
Health worker motivation scores	88.8	86.0	90.5
Training in the past 12 months	72.2	58.6	76.9
**Domain C: Service capacity**			
Basic Infrastructure index	76.9	76.4	75.8
Basic equipment index	84.0	67.1	65.0
Laboratory capacity index	76.5	62.5	67.0
Tracer drugs index	87.9	87.6	88.4
Infection control index	82.0	80.2	88.9
**Domain D: Finance**			
Finance index	* **47.6**	**44.0**	**66.7**
**Domain E: Governance domain**	80.1	87.6	88.1
**Domain F: Service provision**			
Service readiness index	69.7	65.6	68.6
Clinical observation index (Children)	* **31.9**	**71.0**	**72.7**
Clinical observation index (Adults)	54.4	34.3	45.7
Service coverage Children index	71.8	73.	76.4
Service coverage Adult index	51.0	52.5	57.1
**Domain: Overall vision:**			
Service satisfaction index by Gender:			
*Male*	74.4	63.6	76.8
*Female*	72.2	72.7	76.1

* *The mean difference is significant at p<0.05, using ANOVA.*

**Table 5:** Baseline Performance Stratified by Residence in the Six Health System Domains.

Domain	Residence mean scores		
Domain A: Patients and community:	Peri urban	Rural	Hospital
Patient satisfaction children index	63.5	62.2	63.0
Patient satisfaction Adult index	70.6	72.8	75.5
**Domain B: Human resources**			
Health worker motivation scores	86.7	88.6	87.7
Training in the past 12 months	50.0	74.3	66.7
**Domain C: Service capacity**			
Basic Infrastructure index	78.8	76.2	73.1
Basic equipment index	72.5	76.3	70.0
Laboratory capacity index	81.3	67.6	68.8
Tracer drugs index	87.2	87.4	97.0
Infection control index	76.4	83.0	100
**Domain D: Finance**			
Finance index	* **42.9**	**53.9**	**16.7**
**Domain E:Governance**			
Governance index	83.4	84.1	82.1
**Domain E: Service provision**			
Service readiness index*	64.1	68.7	76.5
Clinical observation index( Children)	53.8	50.0	70.0
Clinical observation index (Adults)	42.5	46.5	55.6
Service coverage Children index	71.7	73.2	81.3
Service coverage Adult index	57.8	51.2	51.5
***Domain: Overall vision:***			
Service satisfaction index by Gender:			
*Male*	64.2	73.2	77.5
*Female*	73.3	72.6	75.0

* *The mean difference is significant at p<0.05, using ANOVA.*

When comparing the satisfaction scores by residence, scores were generally lower for children when compared to adults. Across the three districts children's satisfaction scores were below 65%.There was little variation between the residence in children scores with peri-urban and hospital-based health facilities scoring about 63% and rural health facilities scoring 62%. In contrast, adults' scores showed some variation with highest score in hospital based health facilities (75%) and lowest in the peri-urban health facilities (71%). (See Tables 4 and 5).

### Service capacity domain

This domain comprised six indices, each made up from an aggregate of indicators. Across the three study districts the basic infrastructure score was similar at 76%. Basic equipment and laboratory capacity scores showed major variation with Kafue and Luangwa having lower scores when compared to Chongwe. For basic equipment Luangwa scored lowest (65%), followed by Kafue (67%). Chongwe had the highest basic equipment score of 84%, and the laboratory capacity score was lowest in Kafue (63%) and highest in Chongwe (77%). Infection control scores were highest in Luangwa (90%) and lowest in Kafue (80%).

**Figure 1:**
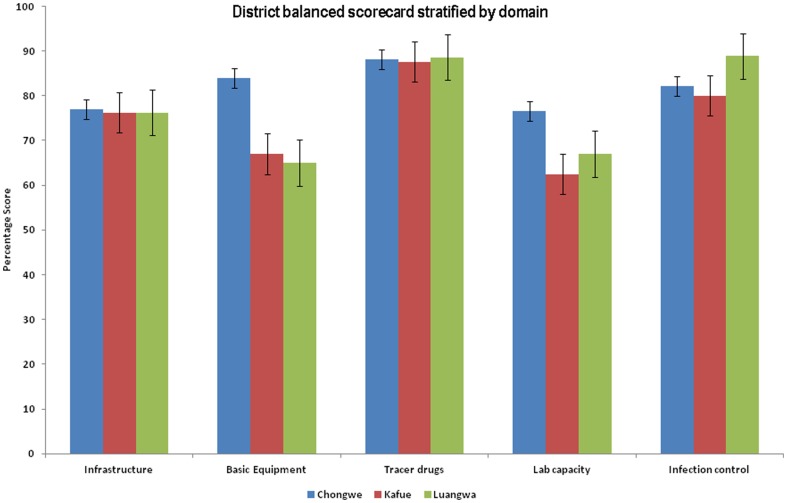
District balanced Scorecard stratified by domain. This figure shows district scores stratified by domain. The domain comprised six indices, each made up from an aggregate of indicators. Across the three study districts the basic infrastructure score was similar at 76%. Basic equipment and laboratory capacity scores showed major variation with Kafue and Luangwa having lower scores when compared to Chongwe. For basic equipment Luangwa scored lowest (65%), followed by Kafue (67%). Chongwe had the highest basic equipment score of 84%, and the laboratory capacity score was lowest in Kafue (63%) and highest in Chongwe (77%). Infection control scores were highest in Luangwa (90%) and lowest in Kafue (80%).

**Figure 2:**
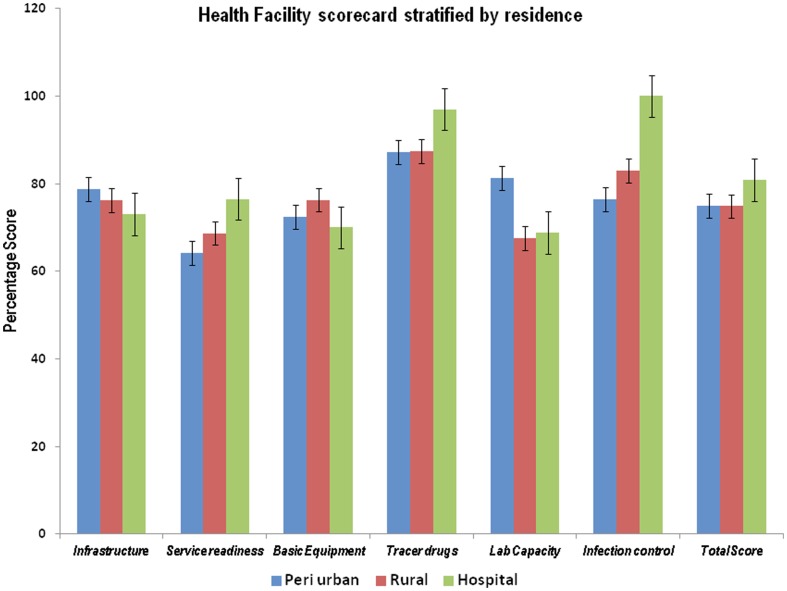
Health facility scorecard stratified by area of residence. This figure shows residential scores which are stratified by domains. It shows that basic infrastructure and basic equipment scores were lowest in hospital-based health facilities (73% and 70% respectively), and the highest scores for basic infrastructure were in the peri-urban health facilities (78%) and rural health facilities for basic equipment (76%). Laboratory capacity had a lower score in rural (68%) and hospital-based health facilities (69%) and was highest in peri-urban health facilities. Infection control was best in hospital-based health facilities (100%) and worst in peri-urban health facilities (76%).Tracer drugs had high scores across the three residential areas (all above 87%).

Tracer drug scores showed little variation across the three districts, all of which scored above 87%. When residential comparisons were made, basic infrastructure and basic equipment scores were lowest in hospital-based health facilities (73% and 70% respectively), and the highest scores for basic infrastructure were in the peri-urban health facilities (78%) and rural health facilities for basic equipment (76%). Laboratory capacity had a lower score in rural (68%) and hospital-based health facilities (69%) and was highest in peri-urban health facilities. Infection control was best in hospital-based health facilities (100%) and worst in peri-urban health facilities (76%).Tracer drugs had high scores across the three residential areas (all above 87%). (See Figures 1 and 2).

### Service provision domain

This domain comprised three indices: firstly whether health facilities offered ten selected essential health services and whether guidelines or protocols were available for each and the availability and recent use of service registers. The second index looked at clinical practice with a focus on under five and adult clinical observations with an overall score for clinical observation for each case observed. The third index looked at community coverage of specific child and adult services. The results showed that scores for this domain across the three study districts were below 80%. Lowest scores for service provision were reported in Kafue (66%) and highest for Chongwe (69%).

Clinical observations showed poor scores for Chongwe (31%) while Kafue and Luangwa showed relatively high scores of 71% and 73% respectively. The differences were statistically significant (p<0.05) Stratified analysis by residence showed that peri-urban health facilities had the lowest scores (64%) while hospital-based health facilities had the highest scores (77%). Adult clinical observations scores were all below 60%. Children's clinical observation score was lowest in rural (50%) and highest in hospital-based health facilities (70%).

Service access coverage score showed no significant difference across the three districts and residence, though adult scores tended to be lower than children scores (Adult range: 51–57%: Children range: 71–82%). (Table 4 and 5).

### Human resources domain

The human resources domain had two separate indicators. One was a measure of the health worker motivation (a composite of 23 items affecting motivation, details are described elsewhere [18] ) and the second was the proportion of interviewed health workers who had received training in the preceding 12 months. The results showed generally high mean scores for motivation across the three study districts (all above 85%).The highest scores were reported in Luangwa (90%) and the lowest scores were noted in Kafue (86%).The proportion of health workers receiving training in the past 12 months was lowest in Kafue (58%) and highest in Luangwa district (77%).

When stratified by residence mean motivation scores remained high across the three residence areas. However, rural residence had a slightly higher mean motivation score when compared to peri-urban or hospital-based health facilities. In terms of training received, peri-urban health facilities had the lowest proportion of health workers who received training (50%).The highest number of health workers receiving training was in rural health facilities at 72%. (Tables 4 and 5).

### Finance system domain

This domain was compiled from three indicators: the availability of a costed action plan (reported or seen), the availability of a person in charge of finance (part or fulltime) and whether the person in charge of finance had received finance training in the last 12 months.

Results showed that Kafue and Luangwa had lower scores in this domain: 44% and 47% respectively, while Chongwe district scored 66%. Wide variation was noted across different residences with hospital-based health facilities scoring lowest at 17%, followed by peri-urban facilities at 43%. Rural health facilities had the highest finance score of 53%). (Table 4 and 5).

### Overall vision

The overall vision was captured by analyzing service satisfaction stratified by gender.

A major gender difference in service satisfaction was noted in Kafue where males showed a lower satisfaction score (64%) when compared to female responders who had a score of 73%.Chongwe and Luangwa showed little variation in scores between males and females. Stratified analysis by residence showed that males in peri-urban health facilities had lower scores when compared to females (male: 64%; female 73%). In both rural and hospital-based health facilities there was a tendency towards males having higher scores when compared to females, but the differences were minimal. (Tables 4 and 5). Linear regression revealed no significant gender differences in adult service satisfaction score after controlling for education status, presenting problem, district and residence. (See Table 6).

**Table 6:** Linear regression model of determinants for Adult service satisfaction score.

	Coeff	Std error	P
(Constant)		6.34	.00
Chongwe	.12	1.90	.12
Luangwa	.19	2.71	*.02
Male sex	.04	2.19	.63
Peri urban	−.04	2.30	.63
Hospital	−.01	4.23	.98
Years in school	.017	.25	.82
**Presenting problem:**			
*Antenatal*	.28	5.93	.20
*HIV treatment*	.01	8.60	.92
*Voluntary Counselling & Testing (VCT)*	.07	13.95	.38
*Tuberculosis Treatment*	.01	14.07	.89
*Malaria/fever*	.01	5.77	.95
*Other services*	.08	5.90	.73

* ***= P<0.05.***

### Linear regression analysis of the association between the different measures of quality of care

Children clinical observation scores were correlated with drug availability (coeff −0.40, p = 0.02) and Chongwe district (coeff −0.43, p = 0.05). The relationship however appeared to be negative meaning that having high drug availability score did not necessarily lead to better clinical care. Chongwe district had a negative association with children clinical observation score suggesting that clinical observations were worse in Chongwe when compared to Kafue which was the reference district. (Model 1). Model 2 shows that adult clinical observation scores were positively associated with adult service satisfaction score (coeff 0.82, p = 0.04) and service readiness (coeff 0.54, p = 0.03), but was negatively associated with motivation scores (coeff −0.40, p = 0.03), meaning that higher motivation score did not necessarily translate into better quality of care. In fact the relationship appeared to be the opposite. Children satisfaction scores were positively associated with governance scores (coeff 0.35, p = 0.05) as shown in model 3.

Models 4, 5, and 6 show no significant association between adult service satisfaction, service coverage (adult and children) with all the independent variables at baseline. (See Table 7).

**Table 7:** Linear regression analysis of the association between the different measures of quality of care.

	Model 1:Dependent variable: Children clinical observation score			Model 2:Dependent variable: Adult clinical observation score			Model 3:Dependent variable: Children satisfaction score			Model 4:Dependent variable: Adult satisfaction score			Model 5:Dependent variable: Adult service coverage score			Model 6:Dependent variable: Children service coverage score		
Variable	coeff	Std err	P	coeff	Std err	P	coeff	Std err	P	coeff	Std err	P	coeff	Std err	P	coeff	Std err	P
(Constant)		129.8	.07		143.03	.93		46.47	.72		32.74	.14		35.20	.08		67.24	.72
Infrastructure Score	−.14	5.45	.49	.22	5.48	.27	.12	1.77	.55	−.27	1.27	.21	−.00	1.35	.95	.37	2.58	.13
Service readiness Score	.44	1.56	.06	.54	1.56	* **.03**	−.38	.48	.08	−.02	.37	.94	.25	.39	.30	−.16	.74	.56
Basic Equipment Score	−.21	5.45	.44	−.49	5.29	.08	.12	1.71	.63	.14	1.26	.64	.40	1.30	.15	−.39	2.49	.22
Drug availability Score	−.40	5.08	* **.02**	.00	5.73	.99	−.09	1.86	.64	.09	1.36	.65	−.07	1.41	.34	.09	2.69	.67
Infection Control Score	.03	5.58	.88	.18	5.59	.34	.23	1.77	.20	−.12	1.33	.54	−.25	1.38	.10	.03	2.63	.82
Governance score	−.06	.66	.77	−.14	.67	.48	.35	.20	* **.05**	−.21	.157	.32	−.08	.17	.70	.02	.32	.92
Training	.06	.24	.71	.23	.23	.17	.11	.08	.49	−.03	.05	.86	−.24	.06	.16	−.05	.12	.79
Peri Urban	.05	14.55	.73	.12	14.81	.45	.20	4.68	.22	−.12	3.51	.50	.18	3.65	.30	−.15	6.96	.44
Hospital	.18	28.36	.28	.04	31.04	.84	.13	10.01	.48	.01	7.42	.99	−.05	7.64	.82	.11	14.59	.61
Chongwe district	−.43	15.99	* **.05**	.26	17.06	.29	.32	5.36	.18	−.12	4.06	.64	−.38	4.20	.13	−.01	8.02	.97
Luangwa District	−.03	16.74	.85	−.08	17.80	.68	−.02	5.80	.99	.26	4.15	.26	.32	4.38	.10	−.07	8.37	.76
Motivation score	.05	1.36	.79	−.40	1.33	* **.03**	.09	.43	.60	.24	.31	.22	.01	.33	.98	−.02	.63	.91
Adult clinical observation score	−.12	.18	.52	-	-	-	.12	.51	.15	.18	1.22	.62	.31	.13	.19	.13	3.33	.76
Children clinical observation score	-	-	-	.45	2.23	.24	−.13	.06	.50	−.02	.05	.92	−.10	.05	.62	−.04	.09	.87
Children satisfaction score	−.11	.59	.56	.041	.60	.84	-	-	-	.35	.14	.11	.18	.15	.39	.23	.28	.34
Adult satisfaction score	.023	.87	.89	.381	.82	* **.04**	.43	1.32	.22	.28	1.22	.67	−.10	.20	.57	.09	.39	.68
Finance score	0.93	20	.77	.315	.21	.08	.07	1.17	.70	.76	2.36	0.76	.27	4.41	.19	.41	.17	.88

* ***P<0.05.***

## Discussion

The study has shown that it is feasible to use a balanced scorecard approach to rank the performance of health facilities and their respective districts. The indicators used in our study are well documented and widely used in low income countries and recommended by WHO for heath facility surveys [13], [19]. We adapted the indicators after extensive consultations with participating districts in order to address the specific Zambian health sector context [20], [21]. The major strength of the study was that we included almost all health facilities in the three study districts apart from pilot sites which made up less than 10% of the total number health of facilities in the study districts.

This work is the first successful application of the balanced scorecard approach to measuring health system performance in Zambia and marks the beginning of an ambitious project to monitor the performance of health system interventions in these target districts for the next four to five years. The methods we used for our study could apply to other health facilities in Zambia with similar rural settings. The evidence generated in this study will help target and adapt the current health system intervention to respond to specific district and health facility needs.

By using a balanced scorecard approach several barriers to providing quality healthcare were highlighted. One important observation was that each district performed well/less well in different domains depending on the residential location of each health facility. This finding emphasises that “one size fits all” interventions may not work well as challenges vary between district and health facility. This means that interventions to strengthen the health system need to be based on current evidence and adapted to suit individual districts and health facilities. In this regard, the use of balanced scorecard approaches or similar tool is essential to monitor the performance and improvements resulting from health system interventions.

In the patient domain, children's service satisfaction scores (based on parent/guardian ratings) were generally lower compared to those of adults' ratings. This could be attributed to the nature of child services which are usually specialised requiring health workers to receive specific training [22], [23]. It could also be due to the fact that more attention is paid when adult patients come for consultation. Service satisfaction scores also varied between districts and residences. Among the three districts, Kafue showed poor scores in both adult and children service satisfaction scores. This could be due to the fact that Kafue is fairly urbanised compared to Luangwa and Chongwe and had a high patient load, which could affect the quality of services and hence the poor scores. It was noted that hospital-based health facilities had better service satisfaction scores compared to rural and peri-urban health facilities. This could be attributed to the availability of qualified health workers and the support given by the mission hospitals to which they were attached. There were at least two clinical officers at each of the hospital-based health facility and referral systems were within the same premises.

Within the human resource domain overall health worker motivation scores were generally high across the study districts and residence. This could be attributed to reporting bias where health workers tended to rate themselves higher than normal as they felt this was desired [24]. Despite this observation there was a tendency towards higher scores for rural health workers when compared to peri-urban and hospital-based health facilities. This is a surprising finding and needs further research as to why rural health workers appeared more motivated when they worked in highly deprived areas where health workers are often unwilling to work. Indeed, motivation and rural origin have been found to be important factors in willingness to work in the rural areas in Rwanda and Ethiopia [25].

Service capacity showed little variation in terms of the availability of basic infrastructure across the three districts. However, substantial variations were noted in the availability of basic equipment and laboratory capacity. Chongwe district scored highly in both indices compared to the two other districts. It is not clear why this was the case but the presence of key partners appeared to favour equipment and laboratory capacity.

The finance domain showed poor scores overall across the three study districts. The data suggest that there are poor financial records and a lack of training in financial management for those in charge of financial record keeping. With the current calls to improve efficiency and accountability in the use of limited resources, there is need to address the deficiencies noted in this domain [26].

Service provision was another domain which showed relatively low scores across the three study districts. Similar findings of low scores at baseline in the service delivery domain were reported in Afghanistan [14]. This finding was not surprising as this domain required health facilities to have guidelines and protocols for various services offered and to actively use them. Physical inspection was used to validate the information given verbally. Most health facilities lacked guidelines and protocols for different health services hence the poor scores recorded in this domain. We therefore recommend that the current health system strengthening intervention gives priority to this domain in order to improve the quality of care given to patients.

Overall vision was captured through gender equity in service satisfaction. There were differences between districts. Generally, males tended to have lower satisfaction scores when compared to females. This could partly reflect the orientation of services towards women and children and less so for men [27]. These gender differences need to be addressed if the vision of equity in access to health care is to be achieved.

There are considerable arguments on what is required to improve quality of service delivery in low income countries. Differential emphasis has been placed on various aspects of quality of care. Some authors have emphasised technical capacities while others have placed emphasis on the process and structural capacities. Which of these is more important still remains a matter of debate and there is conflicting literature. It appears that both technical and structural qualities are important but not sufficient in their own right to improve quality of care [28]. Leonard K et al, 2007 used satisfaction and clinical observations to measure quality of care in rural health facilities in Tanzania. They found that patient satisfaction was correlated with the quality of care based on clinical observations [29]. They did not formally assess process or structural quality. Friedberg M.W et al, 2009 found that structural capacity was correlated with some measures of quality for diabetes but not depressions [30]. Recently Das J et al, 2012 used unannounced standardised patients to measure quality of care in rural and urban India. They found that structural quality such as infrastructure and availability of equipment did not correlate with quality of care and some technical quality measures such as training were weakly correlated with quality of care.

Based on baseline results, our study suggests that both structural and technical capacities could be important for quality improvement and that children and adults measures of quality were sensitive to different measures of process and capacity. In our regression model, we used several dependent outcome variables such as coverage of some essential services in the community, patient clinical observations and service satisfaction and related these to some structural and process capacity indicators. The results were conflicting and highlighted the need for more evidence [28].

The study had a number of limitations which could affect the results of our study. The data was dependent on verbal responses which are prone to information bias [31], [32]. The fact that the respondents were working for the Ministry of Health at the time of interview could have affected the responses with most of the health workers fearing to give a bad image of their institutions for fear of victimisation. This being a cross-section study, it was not possible to attribute the cause and effect among the various domains. Clinical observations have an inherent weakness where those under observation change their usual behaviour thereby giving a false impression about service quality [33].

The balanced scorecard has been criticised as lacking clear focus and promoting multiple goals that might be difficult to reconcile. For example, most balanced scorecard tend to give equal weights to all domains when in reality some domains could be more important than others [34]. In our study, we attempted to give more weight to the clinical observations as this was seen as the most appropriate proxy of quality of care which is the main aim of the study, at least in the short term. Interestingly, there was no correlation between clinical observations and most elements of balanced scorecard. This raises the question of whether the balanced scorecard indicators and domains applied in this study were appropriate and whether the use of the balanced scorecard was serving the intended purpose in our context. We hope to address some of these issues in our follow up study when we compare control and intervention sites. In addition, we will triangulate our data collection to include qualitative methodologies in order to capture some of the important contextual factors and processes that may not be observable in a balanced scorecard.

### Conclusion

The study applied the baseline balanced scorecard to rank the performance of 42 health facilities in three districts of rural Zambia. Differences were noted by district and residence in most domains with finance and service delivery domains performing poorly in all study districts. Despite some limitations, this tool is a useful approach to monitoring health systems intervention in low-income settings and may be valuable in achieving targets towards the health-related Millennium Development Goals.
